# Supplementary material for: Complementary Roles of Two DNA Protection Proteins from Deinococcus geothermalis
Source: Int J Mol Sci. 2022 Dec 27;24(1):469. doi: 10.3390/ijms24010469 (PMC9820295; doi:10.3390/ijms24010469)
Supplement: Supplementary file 1 [file ijms-24-00469-s001.zip › ijms-2056621-supplementary.pdf]

List

Supporting Information.R1.docx 1
Supporting data.R1 3

Supporting information

Table S1. List of primers to construct *D. geothermalis* mutant strains and recombinant plasmids for Dps protein expression.

Primer	Sequence (5′→3′)	RE site
Construction for Δ*dgeo*_0281 mutant strain		
0281_LF_*Kpn*I	5′ AAGGTACCTTCCAGGGGTTCACTT 3′	*Kpn*I
0281_LR_*Sal*I	5′ AAGTCGACCGACTTTCTTCACGCT 3′	*Sal*I
0281_RF_*Xba*I	5′ AATCTAGAAGAATCAAGCTGCGTCCT 3′	*Xba*I
0281_RR_*Pst*I	5′ AACTGCAGCATTGGAAGCGGCATT 3′	*Pst*I
Construction of pCS19_*dgeo*_0257		
pCS19_0257F	5′ AACCATGGCCAAGCGCAGCAAGGTGTT 3′	*Nco*I
pCS19_0257R	5′ AAAGATCTTTGGGACGCGAGGCGTTCCT 3′	*Bgl*II
pCS19_*dgeo*0257_SDM_F*	5′ AGAAATTAACCATG**A**CCAAGCGCAGCAAG 3′	
pCS19_*dgeo*0257_SDM_R*	5′ CTTGCTGCGCTTGG**T**CATGGTTAATTTCT 3′	
Construction of pCS19_*dgeo*_0281		
pCS19_0281F	5′ AACCATGGCAAGAGCCACCAAATCGGCG 3′	*Nco*I
pCS19_0281R	5′ AAGGATCCGTTCATCCGGTCATCGTCCAAGATC 3′	*Bam*HI
pCS19_*dgeo*0281_SDM_F*	5′ GAGAAATTAACCATG**A**CAAGAGCCACCAAA 3′	
pCS19_*dgeo*0281_SDM_R*	5′ ATTTGGTGGCTCTTG**T**CATGGTTAATTTCTC 3′	

* Site-directed mutagenesis (SDM) for second amino acid residue was performed because when we applied *Nco*I for cloning, the first nucleotide of second amino acid was changed to ‘G’. The bold indicates original nucleotide for substitution.

Figure legends

Figure S1. A. Protein profiles from total lysates included both Dgeo_0257 and Dgeo_0281 (DgDps1) by induced different concentrations of IPTG. B. Protein profiles from one step purification of Ni-affinity chromatography both Dgeo_0257 and Dgeo_0281 (DgDps1). Lanes: M. size markers; p, pass through; w, wash; e, elution; conc & nc, concentrated by Viva spin and non-concentrated, respectively; c, control by the purified Dgeo_0257 protein.

Figure S2. Gel filtration of Dgeo_0257 and Dgeo_0281 with/without iron ions. Calibration of gel filtration with size markers marked with arrows: 1, 440 kDa, ferritin; 2, 75 kDa, conalbumin; 3, 43 kDa, ovalbumin; 4, 29kDa, anhydrase from erythrocytes in inner box. Gel filtration profiles of Dgeo_0257 (A) and Dgeo_0281 (B) with protein only, add Fe (II) 1 mM, and add Fe (III) 1 mM conditions.

Figure S3. Various metals effect on EMSA of DgDps1 (Dgeo_0281) and DgDps3 (Dgeo_0257). A, lead; B, cobalt; C, copper; D, caesium; E, chromate.

Figure S4. A, Comparison of growth pattern among wild-type, Δ*dgeo*_0257, and Δ*dgeo*_0281 mutants on TGY medium. B, Comparison of growth pattern among complementary strains when chloramphenicol of 3 μg/mL was present. C, Viability test by hydrogen peroxide treatment of 80, 100, and 120 mM among wild-type, Δ*dgeo*_0257, Δ*dgeo*_0281 mutants and their complementary strains (Δ*dgeo*_0257/pRADgro_*dgeo*_0257, and Δ*dgeo*_0281/ pRADgro_*dgeo*_0281).

Figure S5. qRT-PCR analysis of *dgeo*_0257 and *dgeo*_0281 genes in complementary strains under 50 mM H_2O_2 stress conditions. ** $p < 0.01$.

Figure S1

A

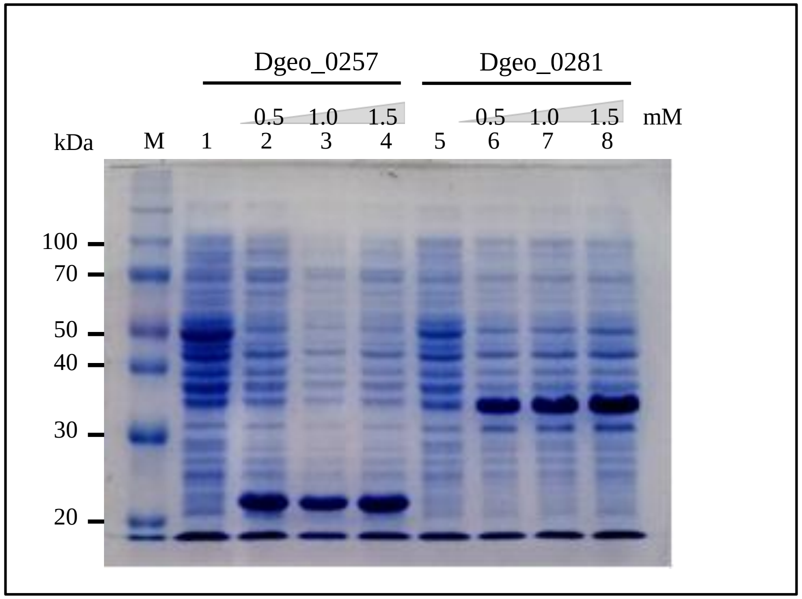

Dgeo_0257
Dgeo_0281
0.5
1.0
1.5
0.5
1.0
1.5
mM
kDa
M
1
2
3
4
5
6
7
8
100
70
50
40
30
20

Figure S1

B

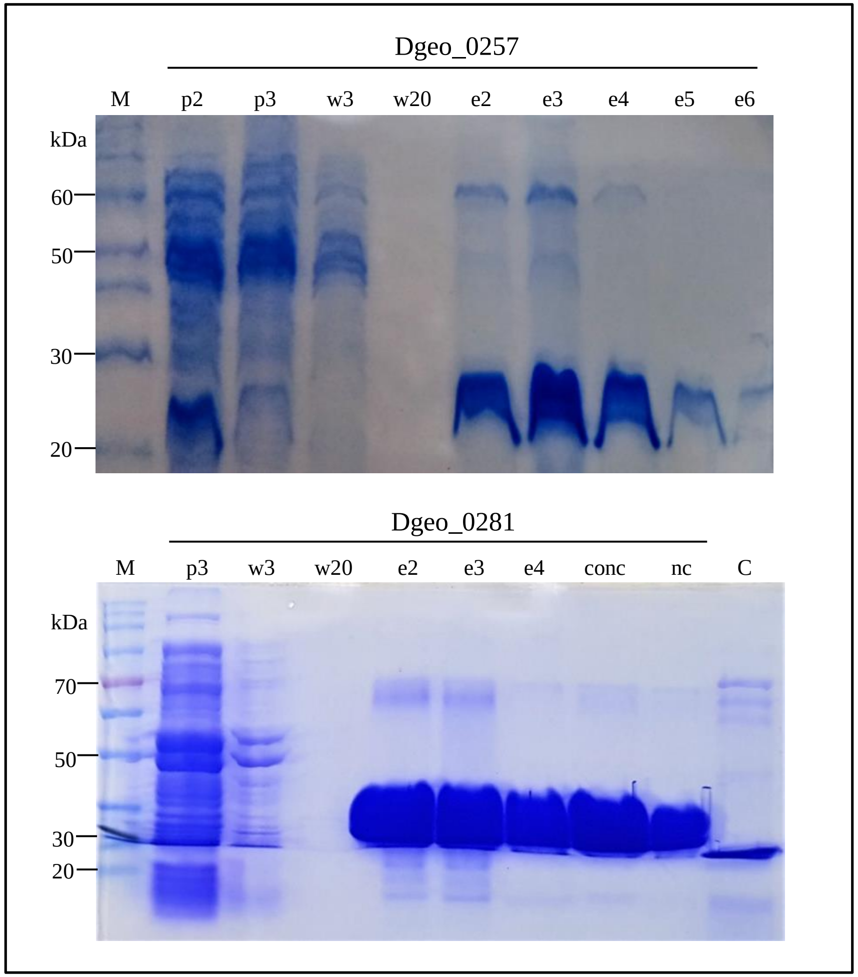
Dgeo_0257
M p2 p3 w3 w20 e2 e3 e4 e5 e6
kDa
60
50
30
20
Dgeo_0281
M p3 w3 w20 e2 e3 e4 conc nc C
kDa
70
50
30
20

Figure S2

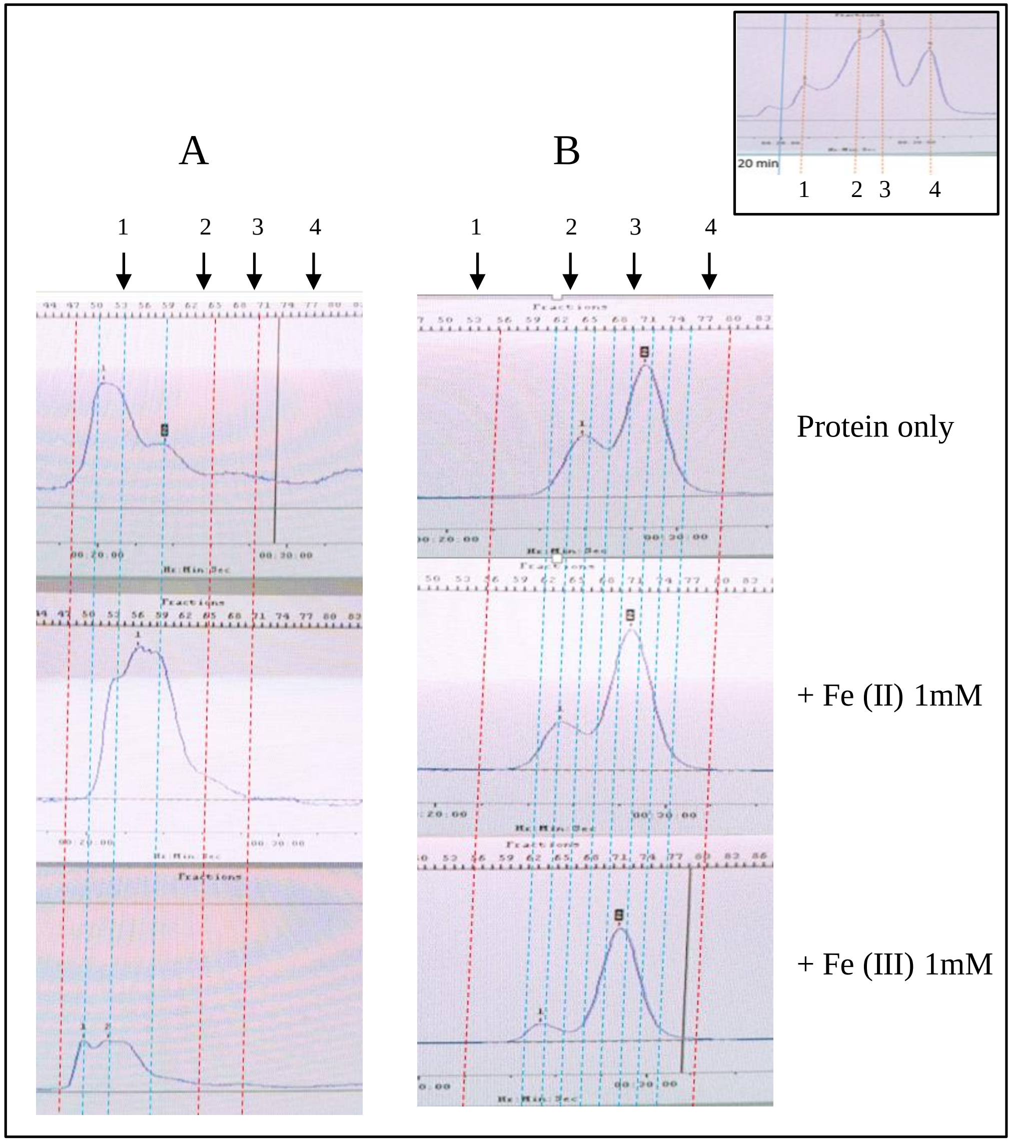

Figure S3

A

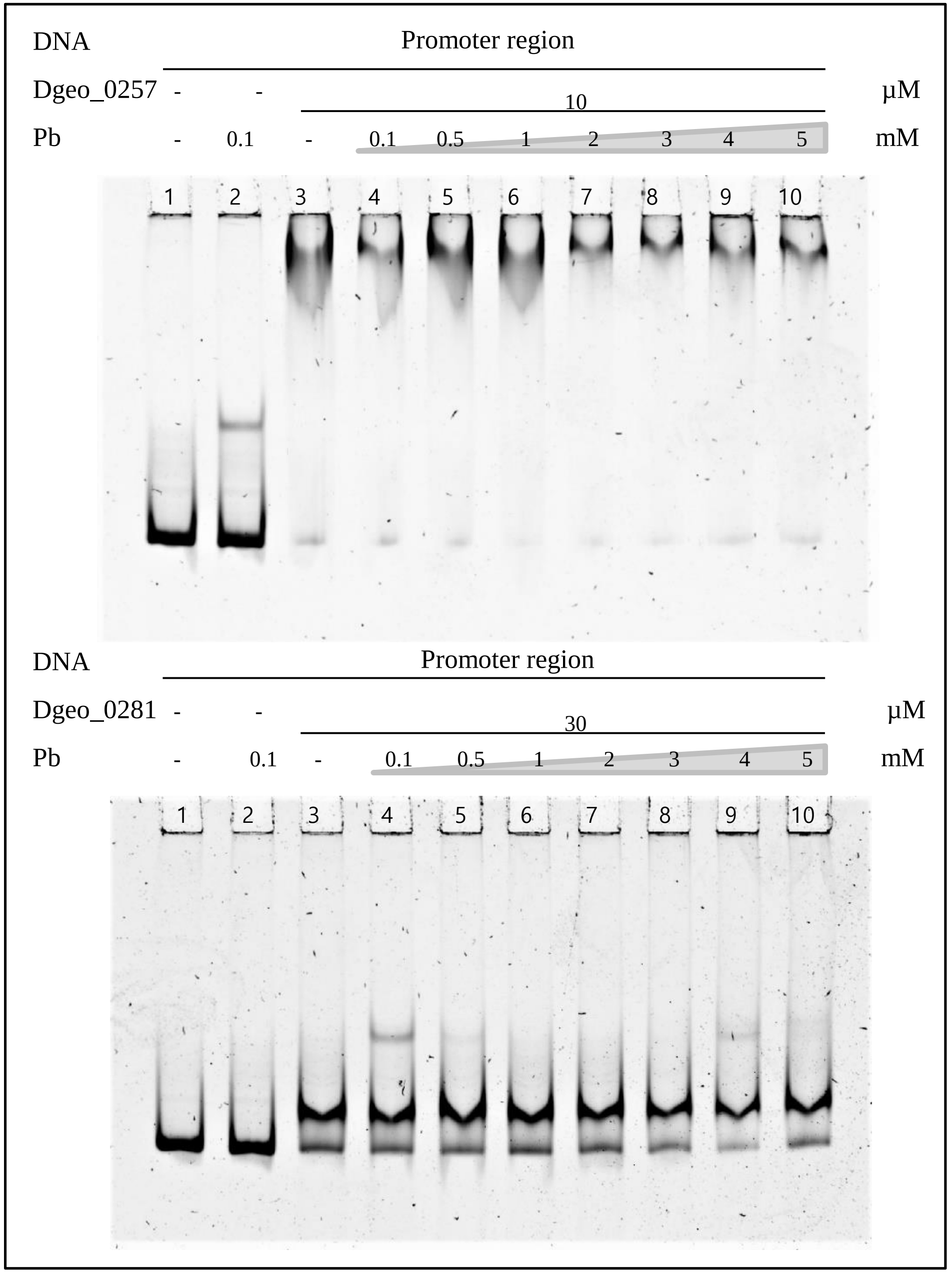

DNA
Promoter region
Dgeo_0257 - -
10
μM
Pb - 0.1 - 0.1 0.5 1 2 3 4 5
mM
1 2 3 4 5 6 7 8 9 10
DNA
Promoter region
Dgeo_0281 - -
30
μM
Pb - 0.1 - 0.1 0.5 1 2 3 4 5
mM
1 2 3 4 5 6 7 8 9 10

Figure S3

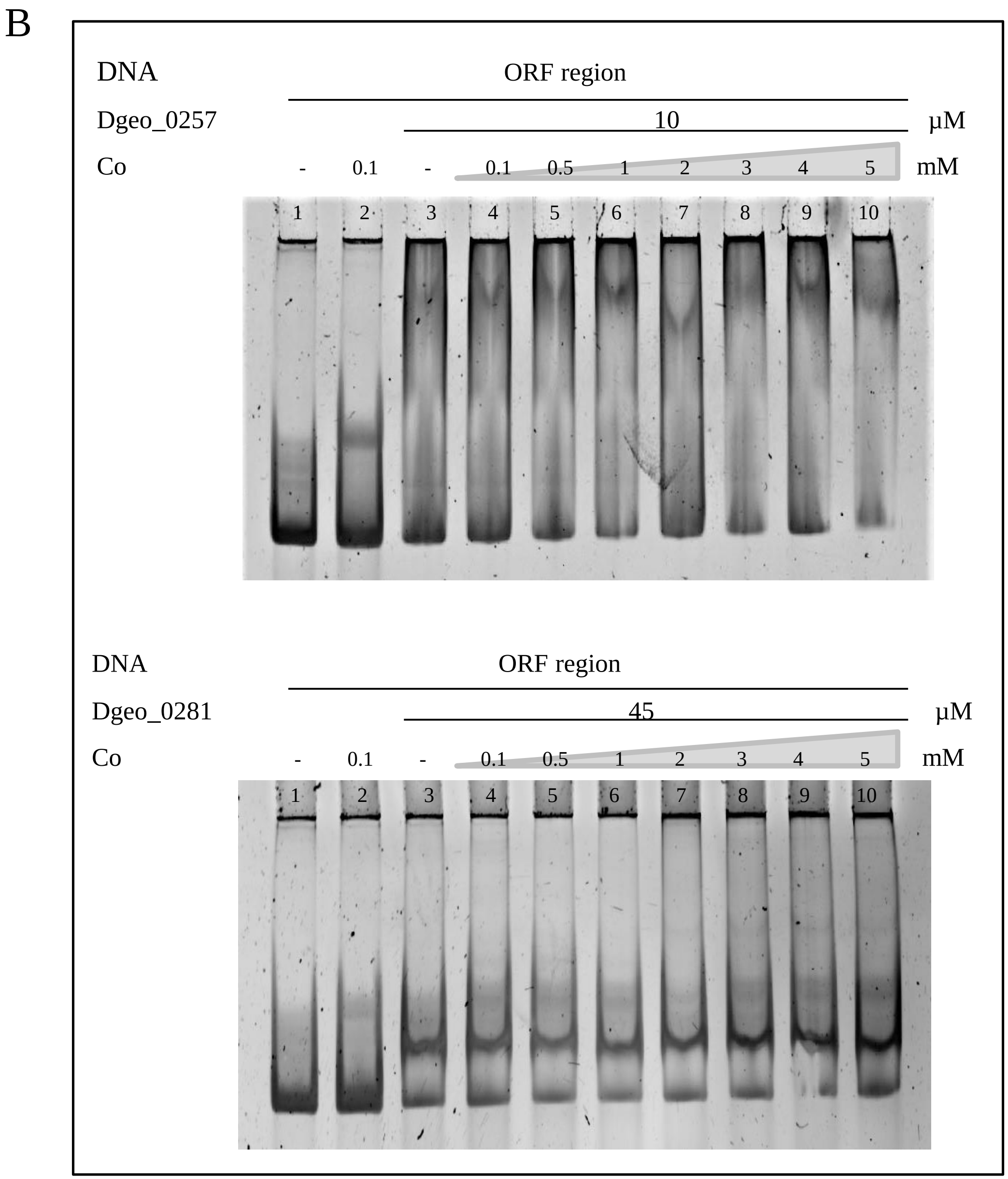
B
DNA
ORF region
Dgeo_0257
10
μM
Co
- 0.1 - 0.1 0.5 1 2 3 4 5
mM
1 2 3 4 5 6 7 8 9 10
DNA
ORF region
Dgeo_0281
45
μM
Co
- 0.1 - 0.1 0.5 1 2 3 4 5
mM
1 2 3 4 5 6 7 8 9 10

Figure S3

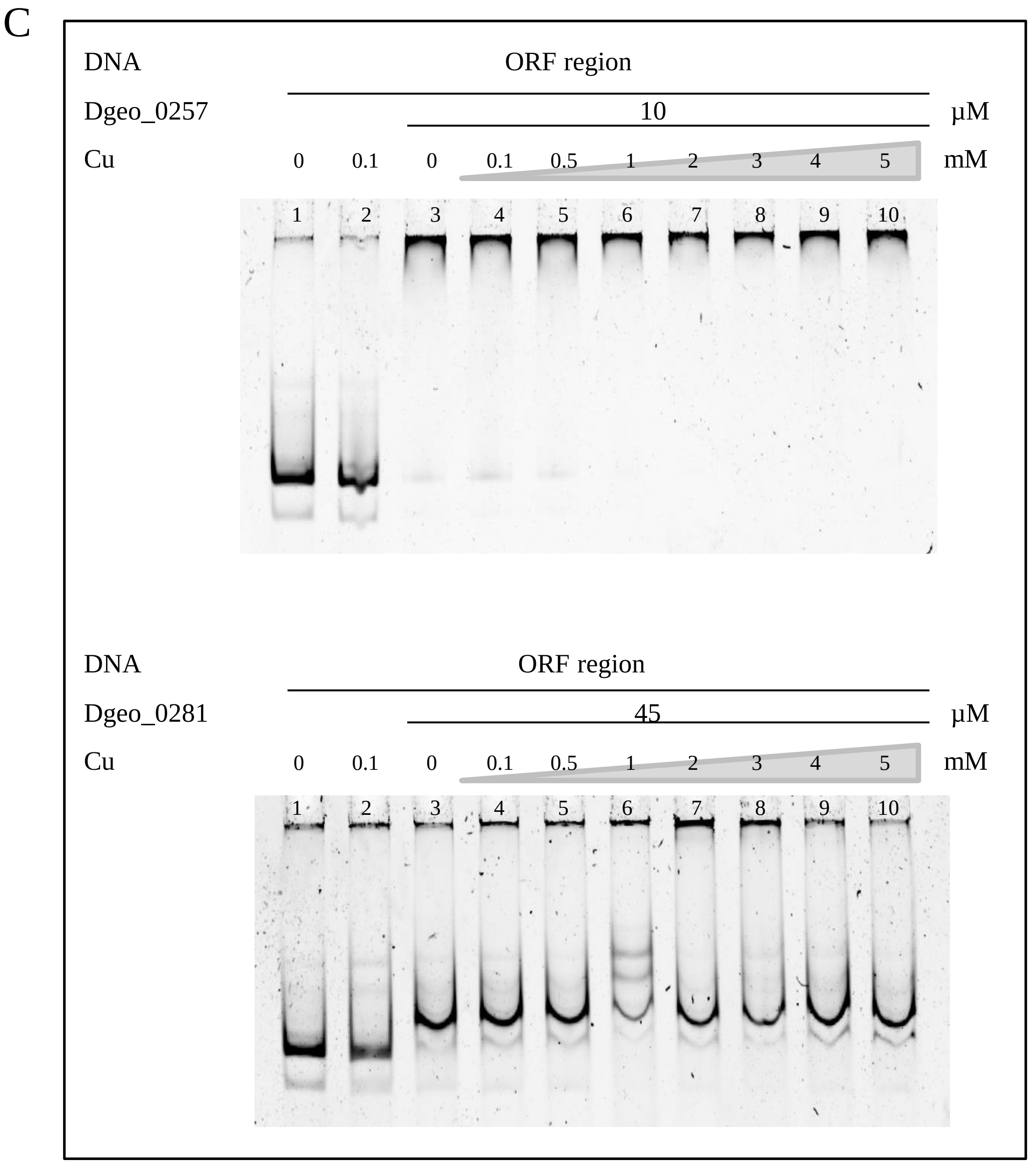
C
DNA
ORF region
Dgeo_0257
10
μM
Cu
0 0.1 0 0.1 0.5 1 2 3 4 5
mM
1 2 3 4 5 6 7 8 9 10
DNA
ORF region
Dgeo_0281
45
μM
Cu
0 0.1 0 0.1 0.5 1 2 3 4 5
mM
1 2 3 4 5 6 7 8 9 10

Figure S3

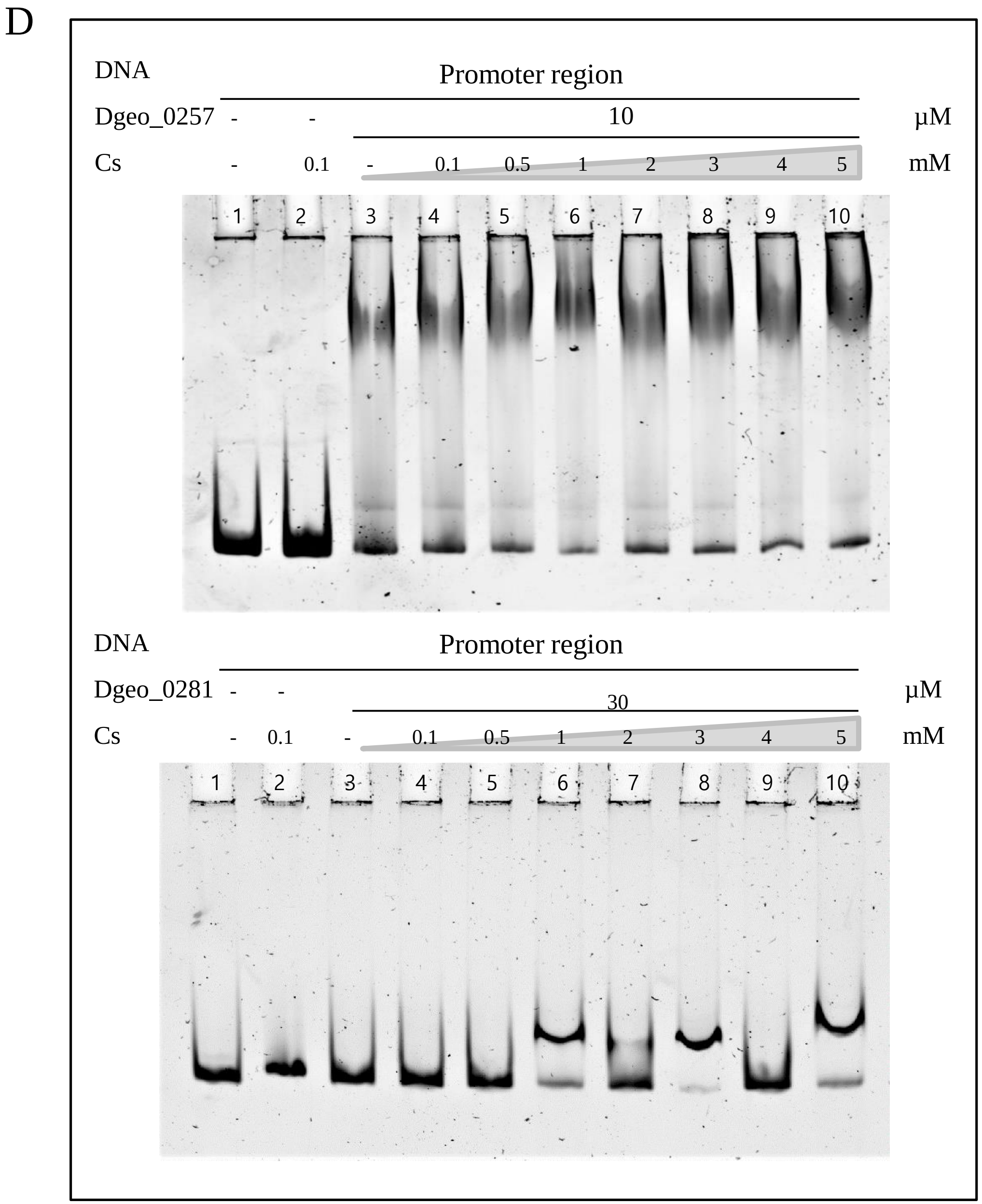
D
DNA
Promoter region
Dgeo_0257 - - 10 μM
Cs - 0.1 - 0.1 0.5 1 2 3 4 5 mM
1 2 3 4 5 6 7 8 9 10
DNA
Promoter region
Dgeo_0281 - - 30 μM
Cs - 0.1 - 0.1 0.5 1 2 3 4 5 mM
1 2 3 4 5 6 7 8 9 10

Figure S3

E

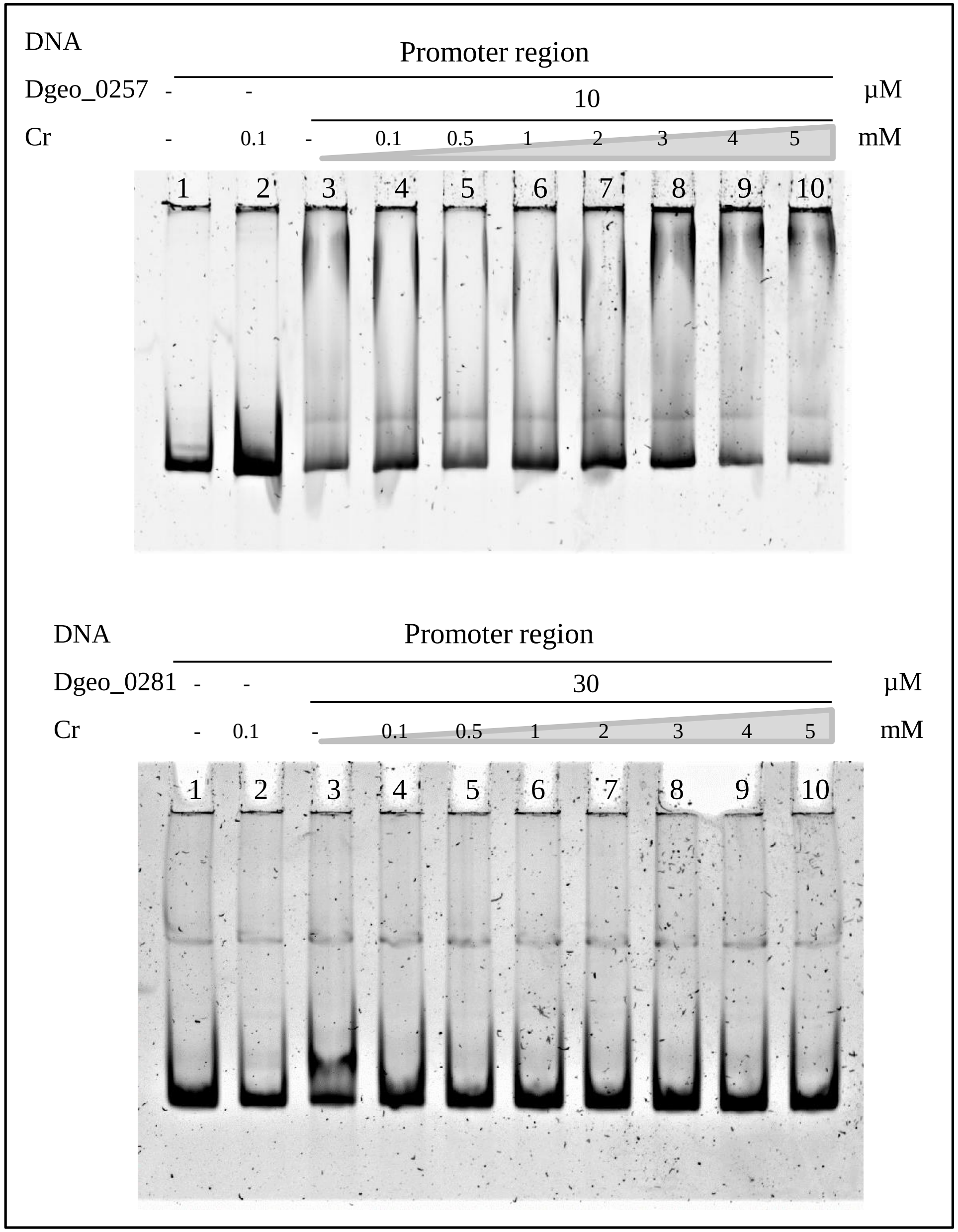

DNA
Promoter region
Dgeo_0257 - - 10 µM
Cr - 0.1 - 0.1 0.5 1 2 3 4 5 mM
1 2 3 4 5 6 7 8 9 10
DNA
Promoter region
Dgeo_0281 - - 30 µM
Cr - 0.1 - 0.1 0.5 1 2 3 4 5 mM
1 2 3 4 5 6 7 8 9 10

Figure S4

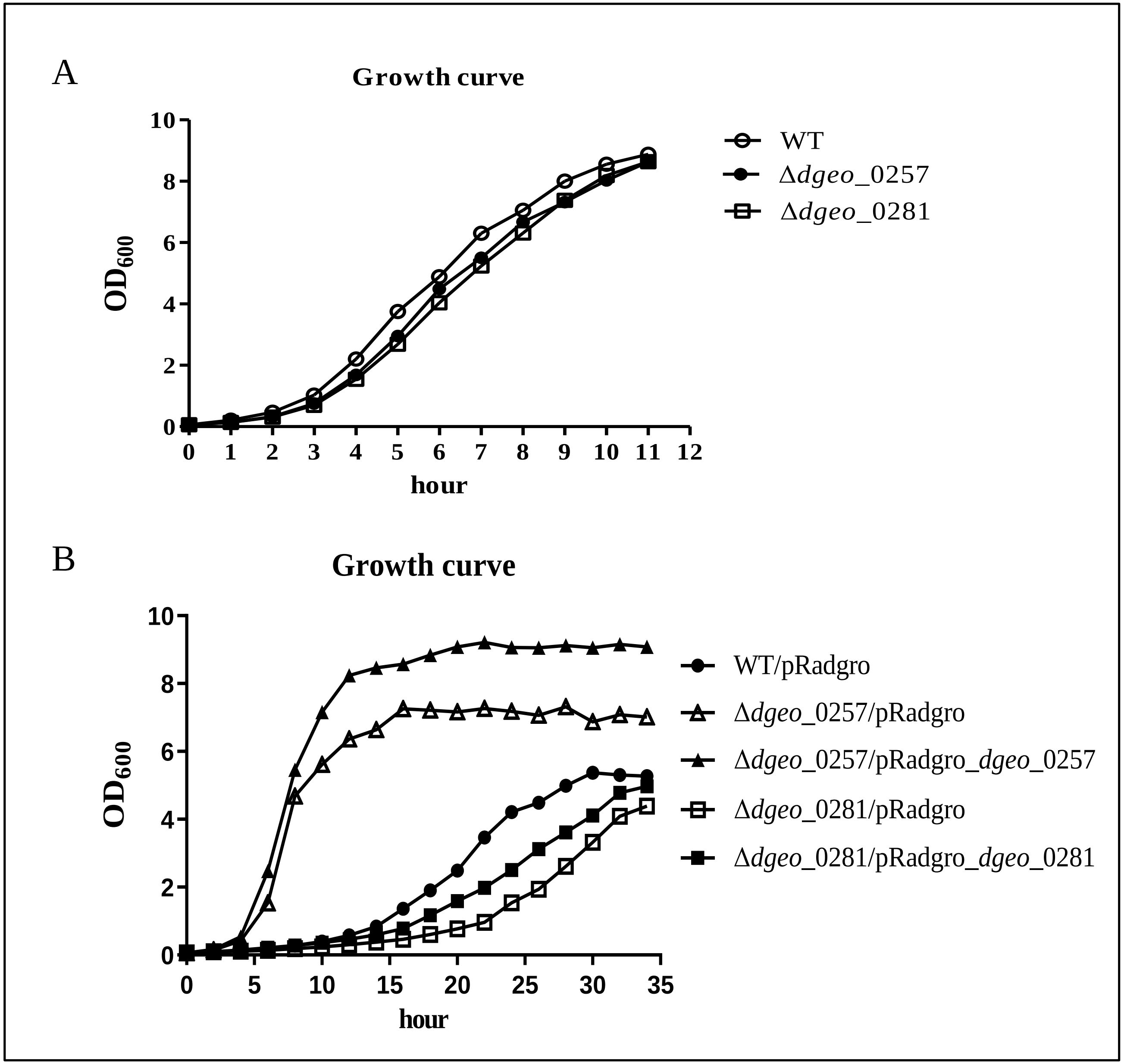

A
Growth curve
OD600
hour
WT
Δdgeo_0257
Δdgeo_0281
B
Growth curve
OD600
hour
WT/pRadgro
Δdgeo_0257/pRadgro
Δdgeo_0257/pRadgro_dgeo_0257
Δdgeo_0281/pRadgro
Δdgeo_0281/pRadgro_dgeo_0281

Figure S4

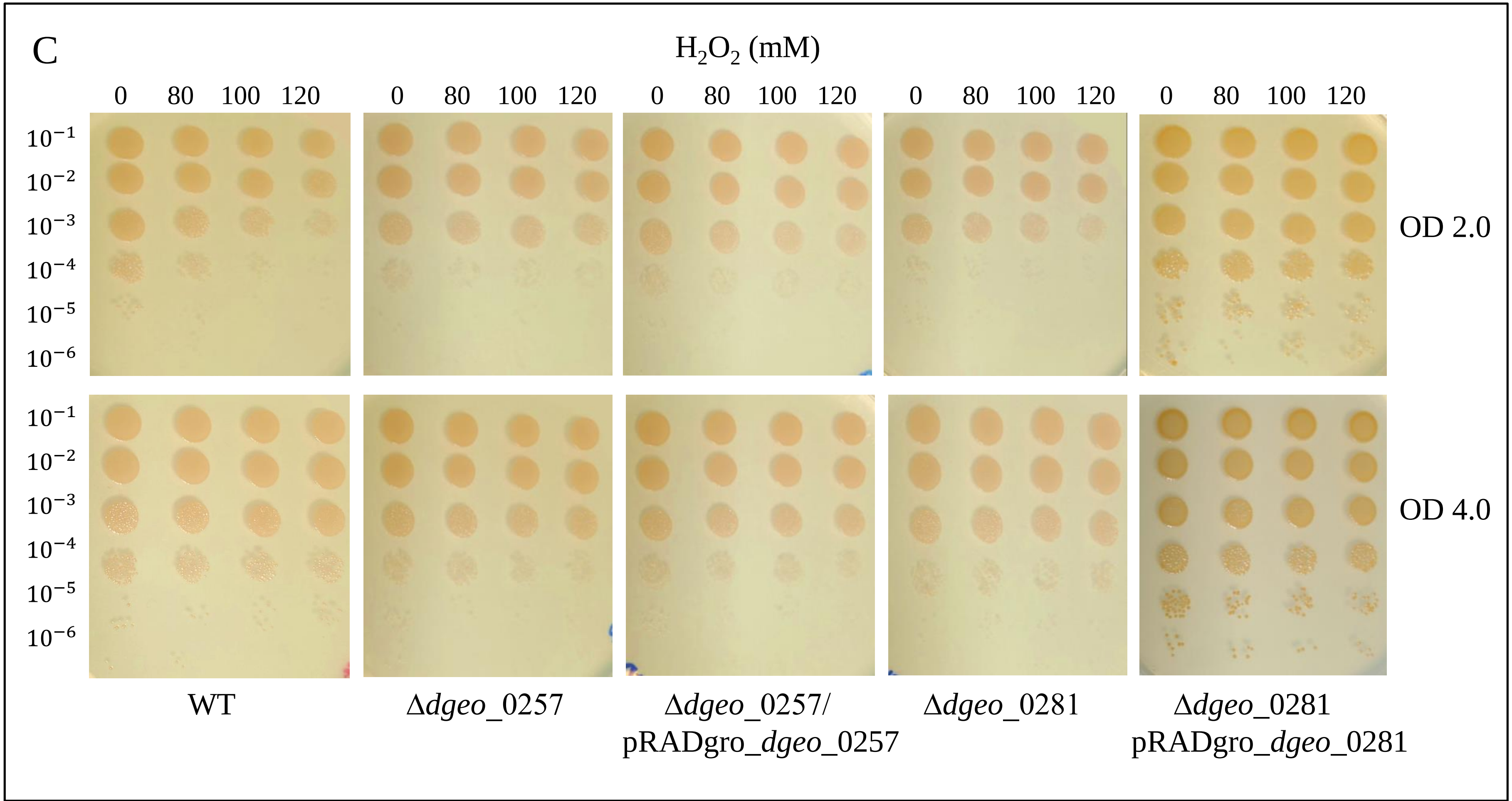
C
H_2O_2 (mM)
0 80 100 120
10^{-1}
10^{-2}
10^{-3}
10^{-4}
10^{-5}
10^{-6}
OD 2.0
OD 4.0
WT
Δ*dgeo*_0257
Δ*dgeo*_0257/ pRADgro_*dgeo*_0257
Δ*dgeo*_0281
Δ*dgeo*_0281 pRADgro_*dgeo*_0281

Figure S5

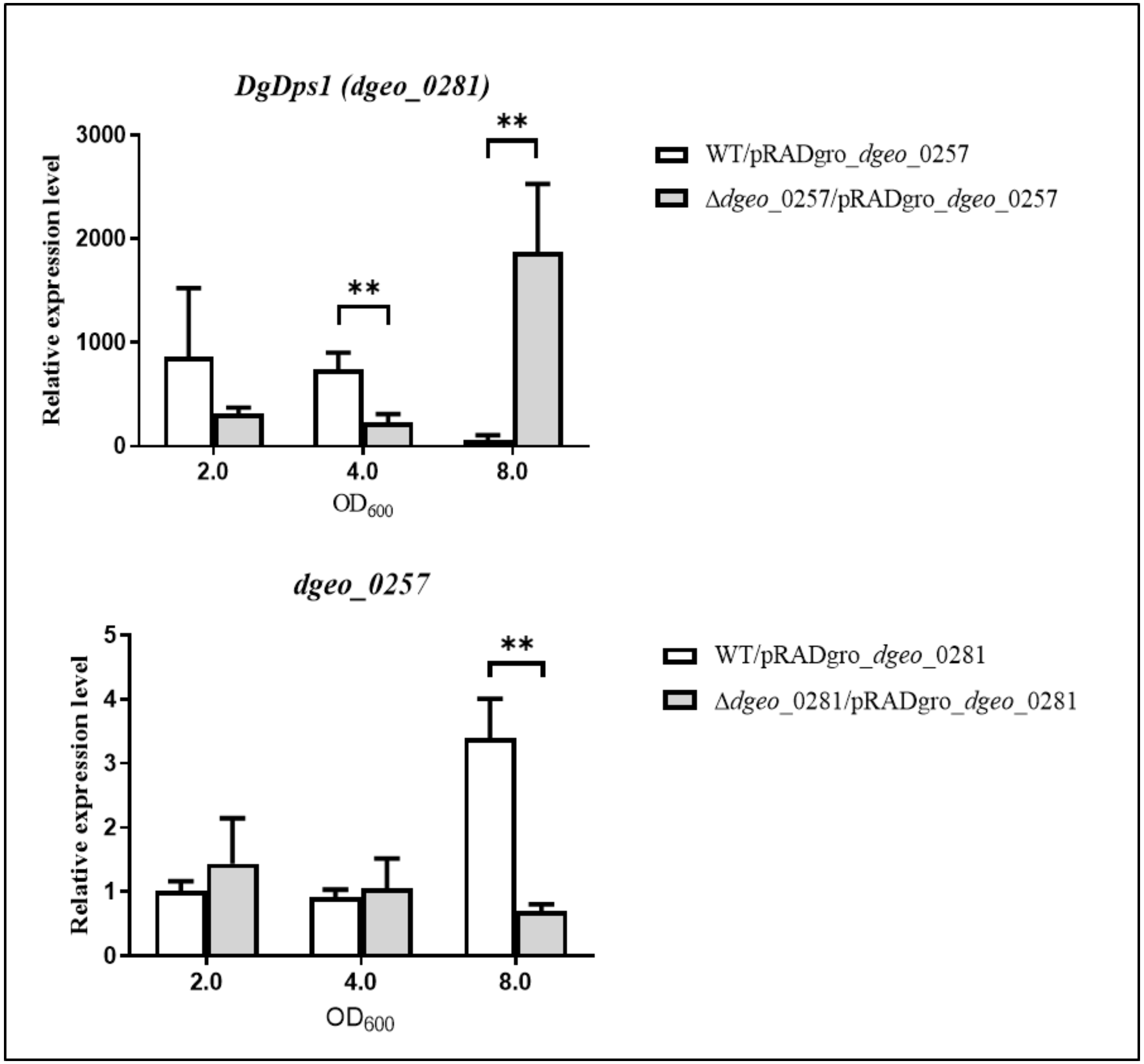
DgDps1 (dgeo_0281)
Relative expression level
3000
2000
1000
0
**
**
2.0
4.0
8.0
OD600
WT/pRADgro_dgeo_0257
Δdgeo_0257/pRADgro_dgeo_0257
dgeo_0257
Relative expression level
5
4
3
2
1
0
**
2.0
4.0
8.0
OD600
WT/pRADgro_dgeo_0281
Δdgeo_0281/pRADgro_dgeo_0281
